# Genome-Wide Identification and Analysis of the VQ Motif-Containing Protein Family in Chinese Cabbage (*Brassica rapa* L. ssp. *Pekinensis*)

**DOI:** 10.3390/ijms161226127

**Published:** 2015-12-02

**Authors:** Gaoyuan Zhang, Fengde Wang, Jingjuan Li, Qian Ding, Yihui Zhang, Huayin Li, Jiannong Zhang, Jianwei Gao

**Affiliations:** 1 College of Horticulture, Gansu Agricultural University, Lanzhou 730070, China; zgy741852@126.com; 2 Shandong Key Laboratory of Greenhouse Vegetable Biology, Shandong Branch of National Vegetable Improvement Center, Institute of Vegetables and Flowers, Shandong Academy of Agricultural Sciences, Jinan 250100, China; wfengde@163.com (F.W.); lijj0620@163.com (J.L.); dingqian87@126.com (Q.D.); zyh_0923@163.com (Y.Z.); lihuayin1@163.com (H.L.)

**Keywords:** VQ motif-containing proteins, Chinese cabbage, expression patterns

## Abstract

Previous studies have showed that the VQ motif–containing proteins in *Arabidopsis thaliana* and *Oryza sativa* play an important role in plant growth, development, and stress responses. However, little is known about the functions of the *VQ* genes in *Brassica rapa* (Chinese cabbage). In this study, we performed genome-wide identification, characterization, and expression analysis of the *VQ* genes in Chinese cabbage, especially under adverse environment. We identified 57 *VQ* genes and classified them into seven subgroups (I–VII), which were dispersedly distributed on chromosomes 1 to 10. The expansion of these genes mainly contributed to segmental and tandem duplication. Fifty-four *VQ* genes contained no introns and 50 VQ proteins were less than 300 amino acids in length. Quantitative real-time PCR showed that the *VQ* genes were differentially expressed in various tissues and during different abiotic stresses and plant hormone treatments. This study provides a comprehensive overview of Chinese cabbage *VQ* genes and will benefit the molecular breeding for resistance to stresses and disease, as well as further studies on the biological functions of the VQ proteins.

## 1. Introduction

To survive adverse environmental conditions, plants have evolved a wide range of complex mechanisms to respond to external stimuli [1]. Such responses are controlled by a complex network regulated by transcription factors (TFs) and other cofactors; though the cofactors do not bind DNA like TFs, they could interact with TFs to co-regulate plant transcriptional machinery in response to the surrounding environment [2].

Over the past several years, plant-specific VQ motif–containing proteins, which were named after the highly conserved amino acid sequence “FxxxVQxL/F/VTG”, were found in many monocotyledon and dicotyledon plants [3,4,5,6]. VQ motif–containing proteins can interact with the WRKY TFs [3] and play many important roles in plant growth and development. For example, the N-terminal peptide of *AtVQ8*, predicted to be a chloroplast targeting signal and mutation in this gene, results in pale-green and stunted-growth phenotypes [3]. *IKU1*, also named *AtVQ14*, is expressed in the endosperm during the early stages of seed development and directly regulates endosperm and seed growth [7]. *AtVQ29* is expressed at a higher level in stem than root, rosette leaf, flower, and silique, and its over-expression reduces the hypocotyl growth under the far-red and low intensity of white light conditions [8]. In soybean, *GmVQ1*, *-6* and *-53* are highly expressed during seed development [5].

VQ proteins also have a vital function in resistance to abiotic and biotic stresses. *AtVQ9* was reported to act antagonistically with *WRKY8* to mediate responses to salt stress and decrease the DNA-binding activity of *WRKY8* [9]. AtCaMBP25 (AtVQ15), a novel calmodulin-binding protein, functions as a negative regulator of plant responses to osmotic stress [10]. MKS1, another VQ motif protein (AtVQ21), acts as a substrate for MAP kinase 4 (MPK4) which functions as a regulator of pathogen defense responses, and MKS1 was also found to form complexes with WRKY25 and WRKY33 [11,12]. Nuclear-encoded sigma factor binding protein1, SIB1 and SIB2, which were renamed AtVQ23 and AtVQ16, respectively, can be rapidly and strongly induced by pathogens [13,14] and were found to recognize the C-terminal WRKY domain and stimulate the DNA-binding activity of WRKY33 [14]. Some AtVQ proteins were phosphorylated by mitogen-activated protein kinases (MAPKs), MPK3 and MPK6, and most of these MPK3/6-targeted VQ proteins interacted with WRKY TFs to regulate plant immune responses [15,16].

At present, 34, 39, 74, and 18 *VQ* genes were identified in *Arabidopsis thaliana* [3], *Oryza sativa* [4], *Glycine max* [5], and *Vitis* *vinifera* L. [6], respectively. Chinese cabbage (*Brassica rapa* L. ssp. *pekinensis*), an important vegetable crop known for its high nutritional value, is widely cultivated in Asia. However, to our knowledge, the *VQ* gene family from Chinese cabbage has not been characterized in detail.

In this study, we performed a genome-wide bioinformatics analysis of the VQ motif–containing proteins, including genome locations, evolutionary divergence, and gene structure. In addition, expression patterns of these genes were analyzed by quantitative real-time PCR (qRT-PCR) in different tissues and in response to abiotic stresses and hormone treatments. The detailed information provided in this study will facilitate further research on functional characterization of the *VQ* genes in Chinese cabbage.

## 2. Results

### 2.1. Identification and Sequence Analysis of VQ Genes in Chinese Cabbage

A total of 57 genes encoding highly conserved VQ motif–containing proteins were identified in Chinese cabbage and the sequences were downloaded from the *Brassica* database [17] (Tables S1 and S2). All the *VQ* genes were assigned specific names according to their *A. thaliana* orthologs [18] (Table 1), which were determined based on the instructions of Gramene [19]. If two or more Chinese cabbage genes had the same homologous gene in *A. thaliana*, one additional number was added after their specific name to distinguish them [20]. For instances, Bra007265, Bra014675, and Bra014674 were homologs of AtVQ23, so they were named BrVQ23-1, BrVQ23-2, and BrVQ23-3, respectively. Therefore, of the 57 BrVQs, 56 putative BrVQs were renamed based on the sequence similarity to 29 AtVQ proteins, and the remaining one (Bra006328), whose ortholog was AT5G14640 (shaggy-like kinase 13), was also identified as a BrVQ protein and renamed BrVQ35. Subsequent sequence analysis of these 57 *BrVQ* genes showed that the CDS ranges from 282bp to 1707bp and the predicted protein lengths vary in size from 93 to 568 amino acids (Table 1). The majority of the proteins (50/57; 87.7%) contain 300 amino acids or less, whereas two proteins (3.5%), *BrVQ14-1* and *BrVQ35*, were more than 400 amino acids. This result was similar to previous studies in *Arabidopsis* [3] and rice [4], where 85.3% and 89.7% of VQ proteins contain less than 300 amino acids, respectively. Additionally, sequence analysis showed that the molecular weight of the BrVQ proteins ranged from 10.4 to 63.0 kDa and the theoretical isoelectric point (pI) from 4.67 to 10.53 (Table 1).

**Table 1:** Properties of the Chinese cabbage *VQ* genes and proteins.

Gene Name	Gene Locus	Chr. No.	Strand Direction	Location	CDS	Protein		
						Length (aa)	Mol.Wt. (KDa)	pI
*BrVQ1-1*	Bra025998	A06	−	6,588,703–6,588,999	297	98	10.81	4.75
*BrVQ1-2*	Bra016616	A08	+	19,301,503–19,301,796	294	97	10.92	5.13
*BrVQ3-1*	Bra025892	A06	−	8,759,573–8,760,265	693	230	25.07	8.67
*BrVQ3-2*	Bra012276	A07	+	8,889,633–8,890,172	540	179	19.29	5.1
*BrVQ4*	Bra030082	A07	+	6,712,561–6,713,295	735	244	26.74	9.66
*BrVQ5*	Bra035492	A08	−	7,791,236–7,791,901	666	221	25.40	6.58
*BrVQ8*	Bra033934	A02	−	108,00,121–10,800,534	414	137	15.36	10.19
*BrVQ9-1*	Bra035028	A07	−	21,850,607–21,851,491	885	294	31.58	10.12
*BrVQ9-2*	Bra008356	A02	−	14,998,946–14,999,815	870	289	31.19	10.39
*BrVQ10-1*	Bra008359	A02	+	15,035,463–15,035,777	315	104	11.61	5.83
*BrVQ10-2*	Bra003642	A07	−	14,203,152–14,203,469	318	105	11.69	5.01
*BrVQ10-3*	Bra035035	A07	+	21,882,989–21,883,270	282	93	10.44	4.67
*BrVQ11-1*	Bra035182	A07	+	22,479,008–22,479,511	504	167	18.96	7.96
*BrVQ11-2*	Bra003566	A07	−	13,824,994–13,825,515	522	173	19.66	9.69
*BrVQ11-3*	Bra008473	A02	+	15,858,716–15,859,210	495	164	18.79	8.74
*BrVQ12*	Bra039937	A09	+	31,714,821–31,715,237	417	138	16.09	9.66
*BrVQ14-1*	Bra023004	A03	+	8,183,495–8,184,808	1314	437	48.10	8.58
*BrVQ14-2*	Bra017329	A04	+	15,346,657–15,347,685	1029	342	37.24	10.53
*BrVQ14-3*	Bra005358	A05	−	5,012,814–5,014,478	1185	394	43.13	10.09
*BrVQ15*	Bra016956	A04	−	17,404,492–17,405,181	690	229	24.84	7.87
*BrVQ16-1*	Bra000216	A03	+	9,940,673–9,941,098	426	141	15.57	8.91
*BrVQ16-2*	Bra004604	A05	+	1,001,763–1,002,185	423	140	15.29	4.89
*BrVQ18-1*	Bra004825	A05	+	1,984,033–1,984,575	543	180	20.11	9.24
*BrVQ18-2*	Bra037658	A04	+	18,293,805–18,294,347	543	180	19.98	9.33
*BrVQ19-1*	Bra027262	A05	+	20,006,387–20,007,064	678	225	24.21	9.44
*BrVQ19-2*	Bra021096	A01	+	23,997,911–23,998,561	651	216	23.49	9.33
*BrVQ20*	Bra037588	A01	+	22,145,216–22,146,079	864	287	30.28	6.54
*BrVQ21-1*	Bra037569	A01	+	22,005,523–22,006,176	654	217	22.73	6.05
*BrVQ21-2*	Bra022345	A05	+	182,87,618–18,288,268	651	216	23.06	6.29
*BrVQ21-3*	Bra001716	A03	−	18,018,055–18,018,699	645	214	22.95	6.22
*BrVQ22-1*	Bra023849	A01	+	2,0411,389–20,411,979	591	196	20.61	9.69
*BrVQ22-2*	Bra041035	Scaffold000402	−	11,806–12,390	585	194	20.40	9.89
*BrVQ23-1*	Bra007265	A09	−	28,278,350–28,278,799	450	149	16.52	4.88
*BrVQ23-2*	Bra014675	A04	+	2,158,610–2,159,095	486	161	18.10	5.1
*BrVQ23-3*	Bra014674	A04	+	2,155,783–2,156,247	465	154	17.35	5.17
*BrVQ24-1*	Bra007279	A09	+	28,339,520–28,340,206	687	228	23.79	6.59
*BrVQ24-2*	Bra014665	A04	−	2,098,440–2,099,141	702	233	24.57	8.05
*BrVQ25-1*	Bra007373	A09	+	28,879,454–28,879,999	546	181	20.12	6.19
*BrVQ25-2*	Bra014594	A04	−	1,651,117–1,651,650	534	177	19.56	6.7
*BrVQ26-1*	Bra007505	A09	+	29,533,410–29,533,874	465	154	17.58	7.02
*BrVQ26-2*	Bra014514	A04	−	1,142,901–1,143,341	441	146	16.68	8
*BrVQ26-3*	Bra003400	A07	+	13,026,327–13,029,056	846	281	32.87	7.16
*BrVQ27*	Bra039565	A01	−	11,929,165–11,929,719	555	184	19.83	9.76
*BrVQ28*	Bra013438	A01	+	5,666,359–5,666,979	621	206	23.15	5.37
*BrVQ29-1*	Bra010608	A08	+	15,506,771–15,507,115	345	114	12.72	9.05
*BrVQ29-2*	Bra017849	A03	+	30,916,676–30,917,020	345	114	12.84	9.05
*BrVQ30-1*	Bra010666	A08	+	15,875,832–15,876,719	888	295	32.18	6.18
*BrVQ30-2*	Bra011838	A01	−	282,249–283,121	873	290	31.74	7.94
*BrVQ31*	Bra005995	A03	+	1,534,982–153,5506	525	174	19.11	9.37
*BrVQ32-1*	Bra022063	A02	+	18,984,720–18,985,412	693	230	25.41	10.26
*BrVQ32-2*	Bra024996	A06	−	24,596,158–24,596,844	687	228	25.12	9.99
*BrVQ33-1*	Bra003032	A10	+	5,893,364–5,894,047	684	227	25.28	9.99
*BrVQ33-2*	Bra022675	A02	−	8,093,063–8,093,788	726	241	26.93	9.75
*BrVQ34-1*	Bra024362	A06	+	15,221,647–15,222,738	1092	363	39.11	5.76
*BrVQ34-2*	Bra037806	A09	+	3,657,352–3,658,311	960	319	34.26	5.94
*BrVQ34-3*	Bra031876	A02	+	27,356,488–27,357,465	978	325	35.03	6.65
*BrVQ35*	Bra006328	A03	−	3,034,196–3,038,533	1707	568	62.99	8.89

**Figure 1:**
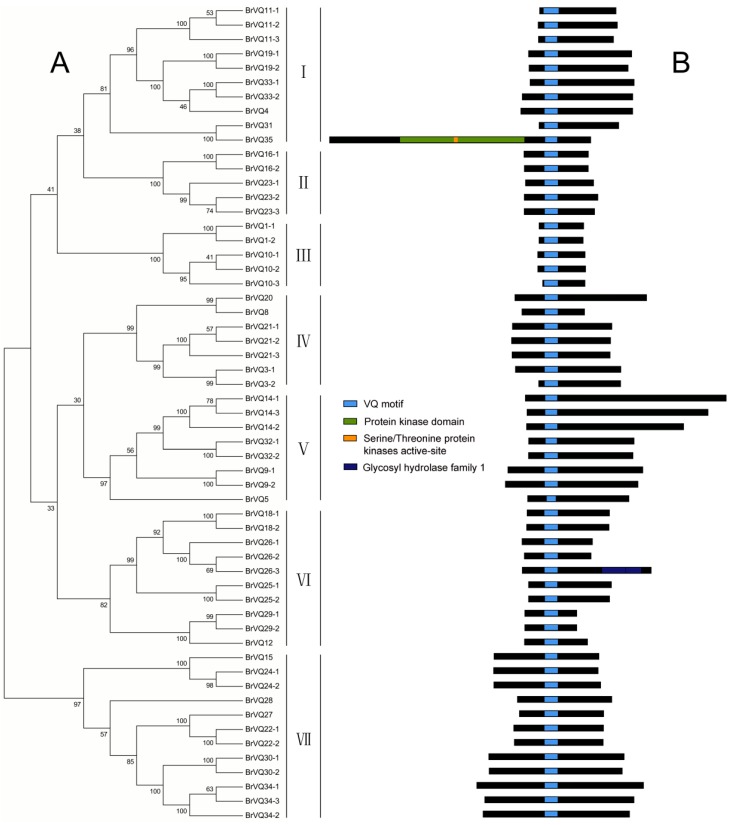
Phylogenetic tree and conserved domain analysis in Chinese cabbage. (**A**) The phylogenetic tree was determined in MEGA5 using the neighbor-joining method with 1000 bootstrap replicates. Based on the clustering of the VQ motif–containing proteins, we classified the proteins into seven groups from subgroup I–VII; (**B**) Domain was analyzed by searching the PlantsP database.

### 2.2. Phylogenetic Tree, Gene Structure, and Conserved Domains Analysis in Chinese Cabbage

**Figure 2:**
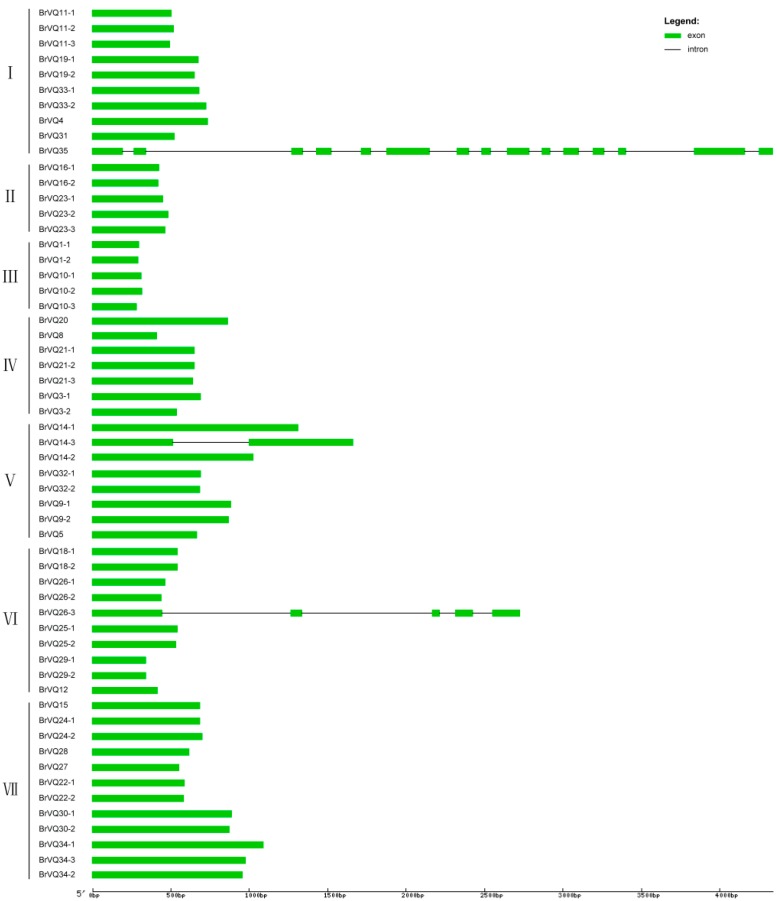
Intron and exon structure of the *VQ* genes in Chinese cabbage. The majority of the *BrVQ* genes only have one exon, except *BrVQ14-3*, *BrVQ26-3*, and *BrVQ35*, which have one, four, and 14 introns, respectively.

**Figure 3:**
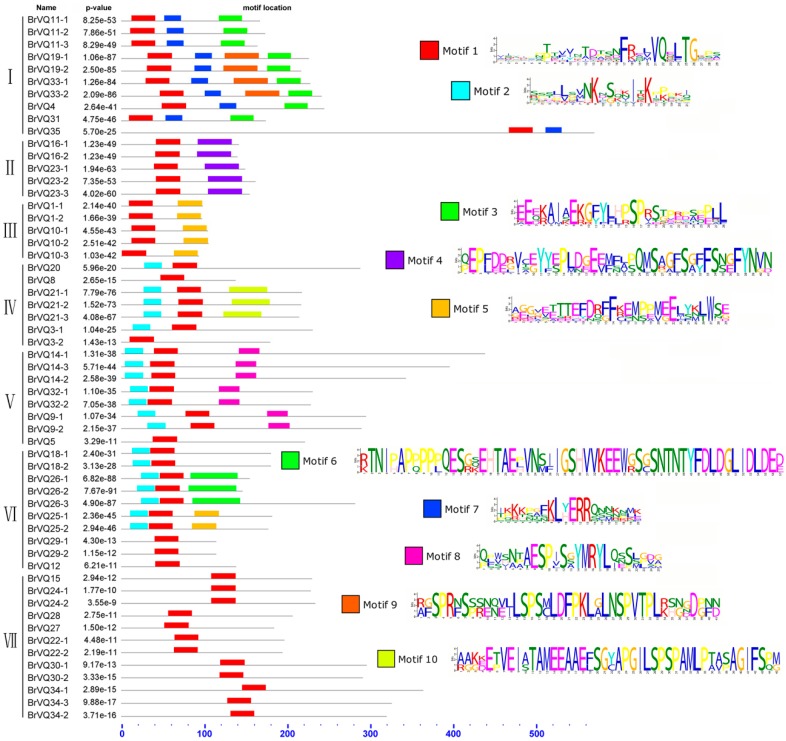
Motif analysis of the VQ proteins in Chinese cabbage. Distribution of the BrVQ conserved motifs in Chinese cabbage was analyzed by the online tool MEME.

A phylogenetic tree was constructed on the basis of the full-length BrVQ protein sequences using the neighbor-joining method. The 57 BrVQ proteins can be divided into seven subgroups, with 10 proteins in subgroup I, five each in II and III, seven in IV, eight in V, 10 in VI, and 12 proteins in subgroup VII, respectively (Figure 1A). In addition, a large number of homologous genes have bootstrap value and alignment identity of more than 70% (Figure 1A, Table S3), implying that the putative *BrVQ* homologous genes have highly similar sequences. Moreover, the gene structure analysis showed that most *BrVQ* genes (54 genes; 94.7%) had no intron, whereas only three genes (*BrVQ14-3*, *BrVQ26-3*, and *BrVQ35*) contained one, four and 14 introns, respectively (Figure 2). The finding is consistent with a previous study [8] where the authors found as many as 30 genes in *Arabidopsis* and 37 genes in rice with no intron. We further performed motif analysis using the PlantsP database and found all BrVQ proteins contain a conserved VQ motif (Figure 1B). The same VQ motif (motif 1) was also detected in all BrVQ proteins when we did an independent analysis using the online tool MEME (Figure 3). By MEME analysis, we also found nine other motifs in the BrVQ proteins, including motif 2 in 19 BrVQ proteins, motif 3 in nine BrVQ proteins, motif 4 in five BrVQ proteins, motif 5 in seven BrVQ proteins, motif 6 in three BrVQ proteins, motif 7 in 10 BrVQ proteins, motif 8 in seven BrVQ proteins, motif 9 in four BrVQ proteins, and motif 10 in three BrVQ proteins, respectively (Figure 3).

### 2.3. Multiple Sequence Alignment and Motif Analysis

**Figure 4:**
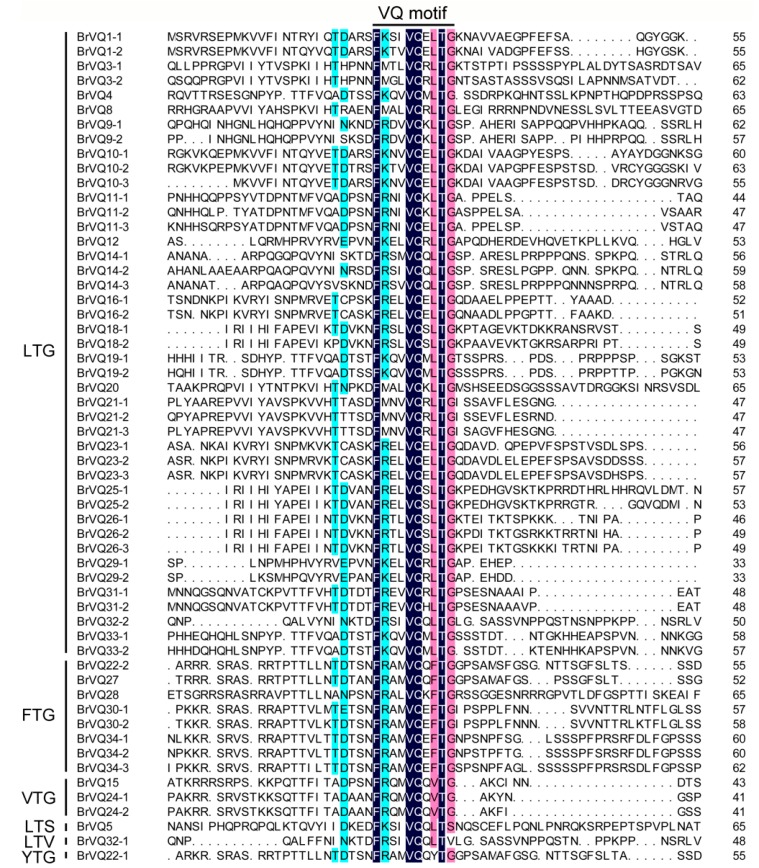
Multiple sequence alignment of the VQ proteins in Chinese cabbage. The sequences were aligned using the DNAMAN software. The highly conserved motif is FxxxVQxLTG.

Multiple sequence alignment was constructed based on the types of *B. rapa* VQ domain proteins (Figure 4). In previous studies, six types of AtVQ proteins (LTG, LTS, LTD, FTG, VTG, YTG) [3] and four types of OsVQ proteins (ITG, LTG, VTG, FTG) [4] were identified. In our study, six types of VQ motifs, including FxxxVQxLTG (43/57), FxxxVQxFTG (8/57), FxxxVQxVTG (3/57), FxxxVQxLTS (1/57), FxxxVQxLTV (1/57), and FxxxVQxYTG (1/57), were identified in Chinese cabbage (Figure 4). Compared to the AtVQ and OsVQ proteins, no BrVQ protein contained the FxxxVQxLTD, FxxxVHxVTG, or FxxxVQxITG motifs, while a unique and conserved sequence “FxxxVQxLTV” was found in the BrVQ32-1 protein.

### 2.4. Chromosome Mapping and Syntenic Analysis of VQ Genes in B. rapa

**Figure 5:**
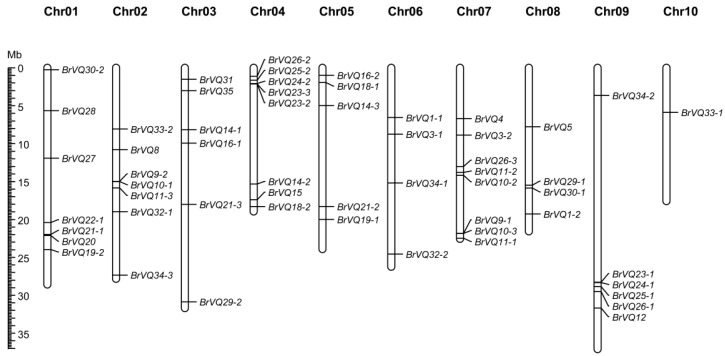
The distribution of the *VQ* genes of Chinese cabbage on 10 chromosomes. The chromosome number is indicated at the top of each chromosome. *BrVQ* gene numbers are shown on the right of each chromosome.

We mapped the physical locations of the *BrVQ* genes on 10 chromosomes of *B.rapa* except one gene, *BrVQ22-2*, which was located on Scaffold000402 (Table 1, Figure 5). Chromosomes 4 and 7 have the highest number of *BrVQ* genes (eight genes each). Chromosomes 1, 2, 3, 9, 5, 6, and 8 contain seven, seven, six, six, five, four, and four *VQ* genes, respectively, whereas chromosome 10 harbors the fewest (only one gene). To get a better understanding of the *BrVQ* gene evolution mechanism, we searched for the syntenic genes and possible *BrVQ* gene duplication events between *A. thaliana* and *B.rapa* with the BRAD program [17,21]. The results suggested that a total of 53 *BrVQ* genes derived from 13 blocks of seven tPCK (translocation Proto-Calepineae Karyotype) chromosomes of the ancestor, respectively, and were distributed on three subgenomes (LF, MF1, and MF2, which stands for less fractionized, more fractionized 1, and more fractionized 2, respectively), including 21 genes on LF, 23 genes on MF1, and 12 genes on MF2 (Table 2). A syntenic relationship between the 53 *BrVQ* genes and the 28 *AtVQ* genes was also detected. However, four genes (*BrVQ3-1*, *BrVQ3-2*, *BrVQ22-2*, and *BrVQ35*) in the *B.rapa* genome had no syntenic relationship with any *A.thaliana VQ* gene. On the other hand, six *AtVQ* genes (*-2*, *-3*, *-6*, *-7*, *-13*, and *-17*) were not in synteny with any *B.rapa* VQ genes. On average, for each *AtVQ* gene, there were one to three copies of a *BrVQ* gene. Seven *BrVQ* loci (the term “locus” instead of “gene” is used here according to the recommendations by Krishnamurthy *et al*. [22]) maintained three copies whereas other *BrVQ* loci maintained either a single copy (13 loci) or two copies (11 loci). Additionally, a total of 41 *BrVQ* genes were detected to have counterparts on segmental duplication, with every member of the segmentally duplicated genes dispersedly distributed on a different chromosome except for four genes (*BrVQ10-2/10-3*, *BrVQ11-1/11-2*) (Figure 5). Interestingly, one tandemly duplicated gene (*BrVQ23-2/23-3*) was found on chromosome 4 (Table 2, Figure 5) with a BrVQ23-2 protein sharing 83% similarity with BrVQ23-3 (Table S3).

**Table 2:** Syntenic *VQ* genes between *Arabidopsis* and Chinese cabbage.

tPCK Chr ^a^	Block	*Arabidopsis* Gene	Chinese Cabbage Gene		
			LF ^b^	MF1 ^c^	MF2 ^c^
tPCK1	A	*AtVQ1*(AT1G17147)	*BrVQ1-1*	*BrVQ1-2*	–
–	–	–	*BrVQ3-1*	–	–
–	–	–	–	–	*BrVQ3-2*
tPCK1	B	*AtVQ4*(AT1G28280)	–	–	*BrVQ4*
tPCK1	B	*AtVQ5*(AT1G32585)	–	*BrVQ5*	–
tPCK6	E	*AtVQ8*(AT1G68450)	–	*BrVQ8*	–
tPCK6	E	*AtVQ9*(AT1G78310)	*BrVQ9-1*	*BrVQ9-2*	–
tPCK6	E	*AtVQ10*(AT1G78410)	*BrVQ10-3*	*BrVQ10-1*	*BrVQ10-2*
tPCK6	E	*AtVQ11*(AT1G80450)	*BrVQ11-1*	*BrVQ11-3*	*BrVQ11-2*
tPCK3	I	*AtVQ12*(AT2G22880)	*BrVQ12*	–	–
tPCK3	J	*AtVQ14*(AT2G35230)	*BrVQ14-3*	*BrVQ14-2*	*BrVQ14-1*
tPCK3	J	*AtVQ15*(AT2G41010)	–	*BrVQ15*	–
tPCK3	J	*AtVQ16*(AT2G41180)	*BrVQ16-2*	–	*BrVQ16-1*
tPCK3	J	*AtVQ18*(AT2G44340)	*BrVQ18-1*	*BrVQ18-2*	–
tPCK2	F	*AtVQ19*(AT3G15300)	*BrVQ19-1*	*BrVQ19-2*	–
tPCK2	F	*AtVQ20*(AT3G18360)	–	*BrVQ20*	–
tPCK2	F	*AtVQ21*(AT3G18690)	*BrVQ21-2*	*BrVQ21-1*	*BrVQ21-3*
tPCK2	F	*AtVQ22*(AT3G22160)	–	*BrVQ22-1*	–
tPCK6	N	*AtVQ23*(AT3G56710)	*BrVQ23-1*	*BrVQ23-2/BrVQ23-3*	–
tPCK6	N	*AtVQ24*(AT3G56880)	*BrVQ24-1*	*BrVQ24-2*	–
tPCK6	N	*AtVQ25*(AT3G58000)	*BrVQ25-1*	*BrVQ25-2*	–
tPCK6	N	*AtVQ26*(AT3G60090)	*BrVQ26-1*	*BrVQ26-2*	*BrVQ26-3*
tPCK4	T	*AtVQ27*(AT4G15120)	*BrVQ27*	–	–
tPCK4	U	*AtVQ28*(AT4G20000)	*BrVQ28*	–	–
tPCK4	U	*AtVQ29*(AT4G37710)	–	*BrVQ29-2*	*BrVQ29-1*
tPCK4	U	*AtVQ30*(AT4G39720)	*BrVQ30-2*	–	*BrVQ30-1*
tPCK5	R	*AtVQ31*(AT5G08480)	–	*BrVQ31*	–
tPCK7	V	*AtVQ32*(AT5G46780)	*BrVQ32-2*	*BrVQ32-1*	–
tPCK5	Wb	*AtVQ33*(AT5G53830)	*BrVQ33-1*	–	*BrVQ33-2*
tPCK7	X	*AtVQ34*(AT5G65170)	*BrVQ34-1*	*BrVQ34-3*	*BrVQ34-2*
–	–	–	–	*BrVQ35*	–

The data were downloaded from the *Brassica* Database [17,21]. ^a^ tPCK Chr: Chromosome of translocation Proto-Calepineae Karyotype, ancestral genome of Brassica species; ^b^ LF: Less fractioned subgenome; ^c^ MFs (MF1 and MF2): More fractioned subgenomes.

### 2.5. Phylogenetic Tree of the VQ Domains in Arabidopsis, Rice and Chinese Cabbage

**Figure 6:**
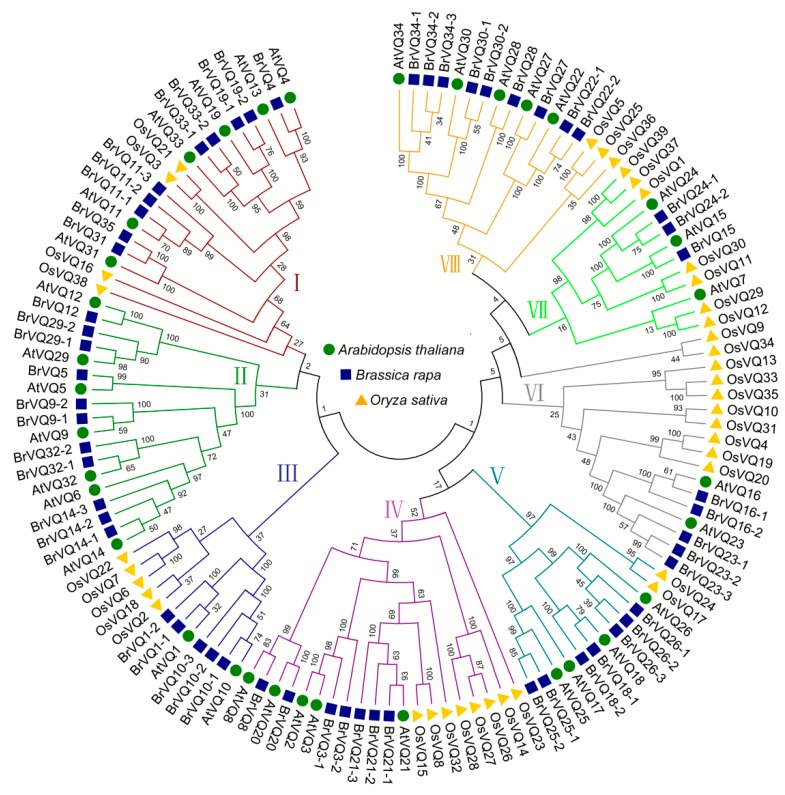
Phylogenetic tree of the VQ motif–containing proteins from *Arabidopsis*, rice, and Chinese cabbage. The tree was determined using the neighbor-joining method with 1000 bootstrap replicates. Based on the clustering of the VQ motif–containing proteins, we classified proteins into eight different groups from Group I to Group VIII. Proteins from *Arabidopsis*, Chinese cabbage, and rice are denoted by green circles, blue squares, and yellow triangles, respectively.

Using the neighbor-joining method, we constructed an un-rooted tree with 34, 57, and 39 VQ amino acid sequences in *Arabidopsis*, Chinese cabbage, and rice, respectively. The sequences were classified into eight groups (I–VIII) (Figure 6). The low bootstrap values in the tree are due to the divergent VQ sequence among the three species. This is not surprising, given that both *A.thaliana* and *B. rapa* belong to cruciferous plants, and the *VQ* genes in these two species were clustered together, while *O. sativa* *VQ* genes clustered by themselves. Moreover, the amino acid sequence of most *VQ* genes in *A. thaliana* and *B. rapa* revealed high similarity of more than 70% with each other (Figure 6, Table S3).

### 2.6. Expression Pattern of the BrVQ Genes in Different Tissues

**Figure 7:**
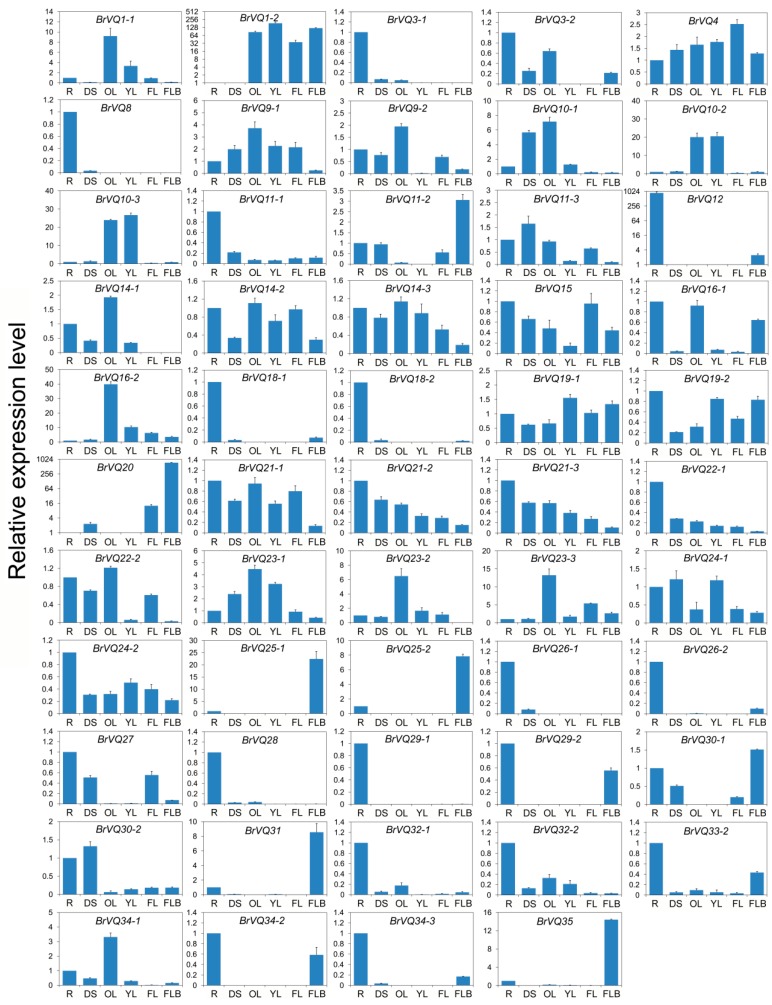
Expression analysis of the *BrVQ* genes in different tissues of Chinese cabbage. The surveyed tissues include root(R), dwarf stem (DS), old leaf (OL), young leaf (YL), flower (FL), and flower bud (FLB). Expression levels of the *BrVQ* genes were normalized to those of *BrActin* and shown relative to their expression in R, except for *BrVQ1-2* and *BrVQ12*, whose expression level was relative to that in DS. The 2^−ΔΔCt^ method was used to calculate the expression levels of target genes in different tissues. The expression levels of *BrVQ1-2*, *BrVQ12*, and *BrVQ20* are shown by log2.

To explore the possible roles of the *BrVQ* genes in Chinese cabbage growth and development, we performed qRT-PCR expression analysis in six tissues, including root (R), dwarf stem (DS), old leaf (OL), young leaf (YL), flower (FL), and flower bud (FLB). Expression of 54 *BrVQ* genes were detected while the other three *BrVQ* genes (*5*, *26-3*, *33-1*) were either absent or poorly expressed. Expression patterns varied among the 54 *BrVQ* genes (Figure 7). For example, 26 *BrVQ* genes, including *BrVQ3-1*, *3-2*, *8*, *11-1*, *12*, *15*, *16-1*, *18-1*, *18-2*, *19-2*, *21-1*, *21-2*, *21-3*, *22-1*, *24-2*, *26-1*, *26-2*, *27*, *28*, *29-1*, *29-2*, *32-1*, *32-2*, *33-2*, *34-2*, and *34-3*, showed higher expression levels in the R than in other tissues; 13 *BrVQ* genes (*1-1*, *9-1*, *9-2*, *10-1*, *14-1*, *14-2*, *14-3*, *16-2*, *22-2*, *23-1*, *23-2*, *23-3*, and *34-1*) were expressed more in the OL than in other tissues; seven *BrVQ* genes (*11-2*, *20*, *25-1*, *25-2*, *30-1*, *31*, and *35*) were expressed mainly in the FLB; four *BrVQ* genes (*1-2*, *10-2*, *10-3*, and *19-1*) were expressed mainly in the YL; three *BrVQ* genes (*11-3*, *24-1*, and *30-2*) were expressed mainly in the DS and only the *BrVQ4* gene was expressed in the FL.

Additionally, some paralogs showed similar expression patterns in different tissues. For example, *BrVQ10-2*/*10-3* had higher expression levels in both OL and YL. *BrVQ21-2*/*21-3* showed similar expression tendency in all tissues. On the contrary, some exhibited different expression patterns in different tissues, including *BrVQ22-1*/*22-2*, *BrVQ24-1*/*24-2*, and *BrVQ30-1*/*30-2*.

### 2.7. Expression Analysis of the BrVQ Genes under Abiotic Stresses

**Figure 8:**
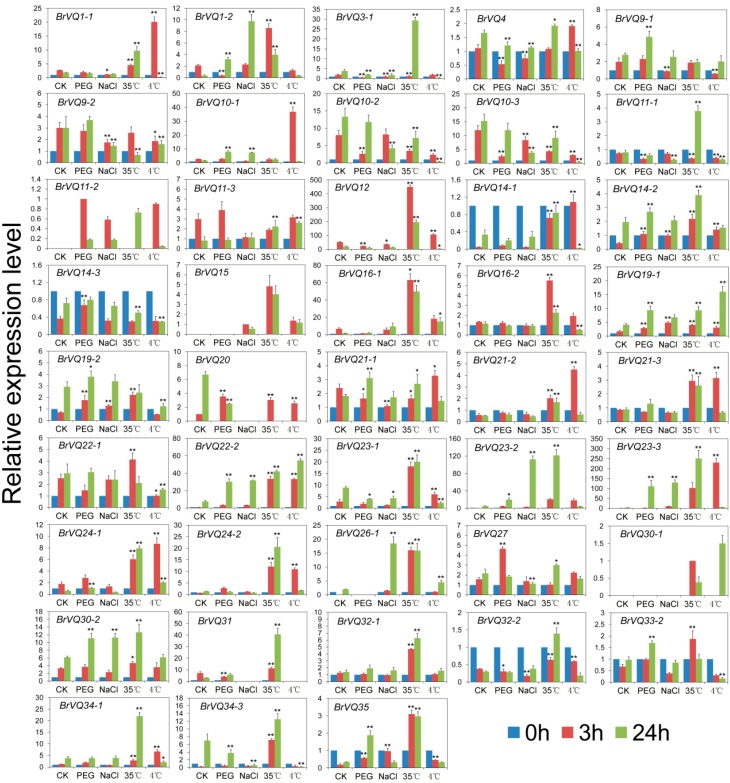
Expression analysis of the *BrVQ* genes under abiotic stresses. Three-week-old plants were treated with 20% (*w*/*v*) PEG_6000_, 20 mmol/L NaCl, 35 °C, and 4 °C for 0, 3, and 24 h before the mature leaves were harvested. Expression of the *BrVQ* genes were normalized to those of *BrActin* and shown relative to the expression of CK at 0 h, except for four *BrVQ* genes (*11-2*, *15*, *20*, *30-1*), whose expression levels were related to the expression of CK treated with PEG, NaCl, CK, 35 °C at 3 h, respectively. The 2^−ΔΔ*C*t^ method was used to calculate the expression of target genes in different tissues. * indicated that the expression level is significantly different from the value of the control (* *p* < 0.05, ** *p* < 0.01).

To further understand the possible roles of the *BrVQ* genes in response to abiotic stresses, we investigated their expression levels under the most common osmotic (polyethylene glycol, PEG_6000_), salt (NaCl), heat (35 °C), and cold (4 °C) stress. Altogether, 43 *BrVQ* genes displayed differential expression compared to the untreated control after at least one stressor treatment (Figure 8), while the expression patterns of the other 14 genes were not detectable. For example, after PEG_6000_ treatment, four (*14-2*, *19-2*, *20*, and *27*) and six genes (*1-2*, *10-1*, *19-1, 22-2*, *23-2*, and *23-3*) were up-regulated whereas seven (*1-2*, *3-1*, *4*, *10-2*, *10-3*, *11-1*, and *12*) and two genes (*20* and *23-1*) were down-regulated more than two-fold compared to the untreated control at 3 h and 24 h of treatment, respectively. *BrVQ35* was up-regulated more than two-fold at both 3 h and 24 h. During salt stress, four genes (*14-2*, *19-1*, *19-2*, and *35*) and five genes (*1-2*, *22-2*, *23-2*, *23-3*, and *26-1*) were significantly induced at 3 and 24 h. On the contrary, five genes (*1-1*, *3-1*, *9-2*, *10-3*, and *23-1*) were down-regulated at 3 h and their expression levels were further decreased two-fold at 24 h with the exception of *BrVQ1-1*. For 35°C treatment, 27 genes were rapidly up-regulated at 3 h. Among these genes, 19 genes (*1-2*, *12*, *16-1*, *16-2*, *21-2*, *21-3*, *22-2*, *23-2*, *23-3*, *24-1*, *24-2*, *26-1*, *30-2*, *31*, *32-1*, *32-2*, *34-1*, *34-3*, and *35*) showed the highest expression levels at 3 h and 24 h. In contrast, three genes (*9-2*, *10-2*, and *10-3*) showed a trend of down-regulation from 3 h to 24 h. Interestingly, *BrVQ3-1* and *BrVQ11-1* were initially down-regulated about two-fold at 3 h but up-regulated more than four-fold at 24 h. In the case of low temperature treatment, five genes (*10-1*, *14-2*, *21-2*, *21-3*, and *34-1*) at 3 h and two genes (*19-1* and *26-1*) at 24 h exhibited at least a two-fold increase in expression compared to the untreated control, while two genes (*9-1* and *22-1*) at 3 h and four genes (*3-1*, *14-3*, *19-2*, and *34-3*) at 24 h showed the opposite expression trend. Interestingly, four genes (*1-1*, *12*, *14-1*, and *23-1*) were up-regulated at 3 h and down-regulated at 24 h more than two-fold. Two genes (*22-2* and *24-1*) and two genes (*10-2* and *10-3*) were induced and inhibited more than two-fold at both 3 h and 24 h, respectively. Four genes (*22-2*, *23-2*, *23-3*, and *35*) were induced to a different extent after osmotic, salt, heat, and cold treatments, while two genes (*10-2* and *10-3*) were inhibited under those conditions.

### 2.8. Expression Analysis of the BrVQ Genes under Phytohormone Treatment

**Figure 9:**
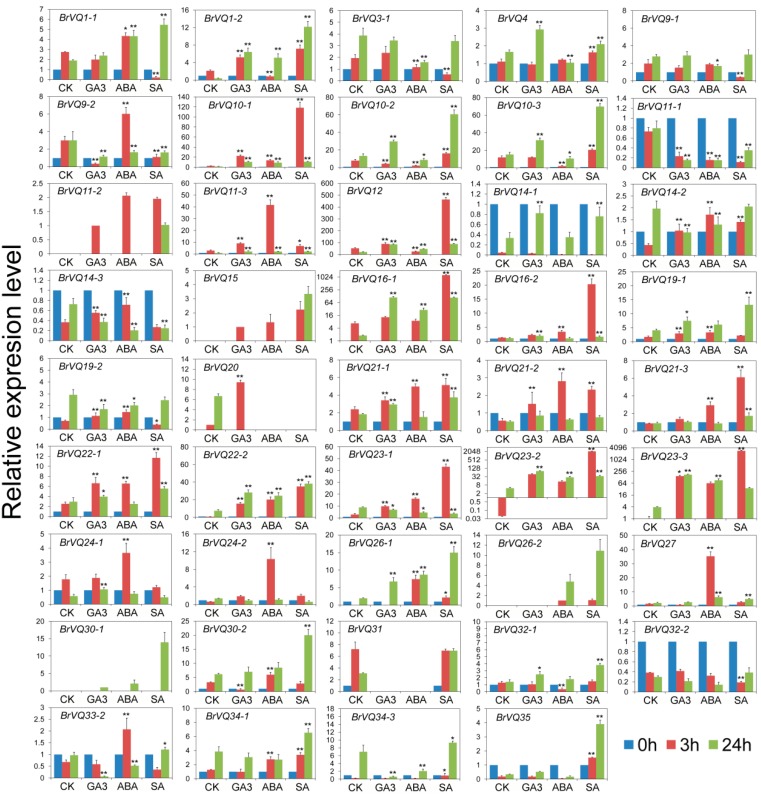
Expression analysis of the *BrVQ* genes under phytohormones. Three-week-old plants were treated with 200 μM GA_3_, 100 μM ABA, and 200 μM SA for 0, 3, and 24 h before the mature leaves were harvested. Expression of the *BrVQ* genes were normalized to those of *BrActin* and shown relative to the expression of CK at 0 h, except for five *BrVQ* genes (*11-2*, *15*, *20*, *26-2*, *30-1*), whose expression levels were related to the expression of CK treated with GA_3_, GA_3_, CK, ABA at 3 h, and GA_3_ at 24 h, respectively. The 2^−ΔΔ Ct^ method was used to calculate the expression of target genes in different tissues. The expression levels of three *BrVQ* genes (*16-1*, *23-2*, *23-3*) are shown by log2. * indicated that the expression level is significantly different from the value of the control (* *p* < 0.05, ** *p* < 0.01).

More and more studies have demonstrated that plant hormones play important roles in plant growth and defense signaling [23,24,25]. To understand the expression response of the *BrVQ* genes to various plant hormones, we carried out Gibberellin A3 (GA_3_), abscisic Acid (ABA) or salicylic acid (SA) treatments in Chinese cabbage plants. We detected the expression of a total of 44 *BrVQ* genes (Figure 9). Upon GA3 treatment, nine genes (*1-2*, *10-1*, *10-3*, *11-3*, *12*, *21-1*, *22-1*, *22-2*, and *23-3*) were up-regulated at 3 h and 24 h, and two genes (*9-2* and *11-1*) were down-regulated. Additionally, some genes showed significant differential expression at one time-point compared to the untreated controls. For example, at 3 h, *BrVQ30-2* was down-regulated more than four-fold while *BrVQ21-2* was up-regulated at least two-fold. At 24 h, *BrVQ33-2* and *34-3* were down-regulated while *BrVQ23-2* was up-regulated at least 10-fold. In the case of ABA treatment, about 25 *BrVQ* genes were induced at 3 h, and some genes (*16-1*, *19-1*, *22-2*, *23-2*, *23-3*, and *26-1*) maintained the up-regulated trend at 24 h, while several other genes (*9-2*, *14-2*, *23-1*, and *33-2*) showed a down-regulated trend at 24 h. Apart from these, six genes (*1-2*, *10-2*, *10-3*, *11-1*, *12*, and *32-1*) showed down-expression of more than two-fold at 3h, and then, three genes (*10-2*, *10-3*, and *11-1*) kept this tendency up to 24 h, while the other two genes (*1-2* and *12*) changed to an up-regulation tendency at 24 h. For SA treatment, the expression levels of 17 genes were higher than the untreated control at 3 h and 24 h, including *1-2*, *4*, *10-1*, *10-2*, *10-3*, *11-3*, *12*, *21-1*, *21-3*, *22-1*, *22-2*, *23-2*, *26-1*, *27*, *34-1*, *34-3*, and *35*. *BrVQ23-2* and *BrVQ23-3*, in particular, were up-regulated more than one thousand-fold at 3h. *BrVQ9-2* and *BrVQ11-1* were down-regulated two-fold at 3 h and 24 h. The expression of *BrVQ23-1* showed a trend of initial increase at 3 h, followed by a decrease at 24 h, compared to the untreated control; however, *BrVQ1-1* showed the opposite expression trend.

### 2.9. Comparison of the Expression Patterns of BrVQ Genes and Their Orthologs in Arabidopsis

To further compare the expression patterns of *BrVQ* genes and their orthologs in *A.thaliana*, the expression patterns of *AtVQ* genes were extracted from Genevestigator [26,27]. We found that the expression patterns in different tissues between *AtVQ* and *BrVQ* genes were similar except for some individual genes (Table S4, Figure 7); for instance, the transcriptional expressions of *BrVQ8*, *BrVQ12*, *BrVQ28*, and *BrVQ29* were mainly detected in the roots, while *AtVQ8*, *AtVQ12*, *AtVQ28*, and *AtVQ29* genes could be detected in the roots, stem, leaf, and flower. Additionally, most of the orthologs of the VQ genes between *B. rapa* and *A. thaliana* also have similar expression patterns under abiotic and hormone treatment. For example, *AtVQ4*/*BrVQ4* and *-10* were induced by cold and SA treatment, *AtVQ11*/*BrVQ11* were induced by cold and ABA treatment, *AtVQ12*/*BrVQ12*, *-14,* and *-21* were induced by heat, cold, and SA, *AtVQ16/BrVQ16* were induced by salt and SA treatment (Table S5, Figure 8 and Figure 9). In summary, after comparing the expression patterns of *BrVQ* genes and their orthologs in *A. thaliana*, we found most, but not all of them, displayed similar expression trends in some tissues and in their responses to various stresses and hormone stimulus. These results further explained the speculation that *VQ* genes may have similar functions in some aspect in two different species; however, the different expression patterns of VQ ortholog genes between these two species should also be noticed.

## 3. Discussion

Previous studies showed that *VQ* genes play an essential role in plant growth, development, and response to adverse environment [3,7,8,9,10,11,12,13,14]. However, there is little information on the characterization of VQ motif–containing proteins in *B. rapa*. Therefore, the comprehensive analysis of *BrVQ* genes and their expression patterns under various abiotic and hormone treatments could be beneficial to further understanding the mechanisms of influencing plant growth and development as well as applying them to Chinese cabbage molecular breeding.

### 3.1. VQ Gene Duplication in Chinese Cabbage

Genome duplication plays an important role in expanding genome content and diversifying gene function because a duplication event can evolve into genes with new functions [28]. After genome duplication, processes such as nonfunctionalization (duplicated genes are silenced), subfunctionalization (function is partitioned between the new paralogs), and neofunctionalization (duplicated genes gain new functions) generally take place so that genes are either lost or fixed [22,29,30]. *Brassica rapa* is a mesopolyploid crop that has undergone the whole genome triplication (WGT) event since its divergence from *Arabidopsis* *thaliana* [31]. Since there are 34 *VQ* genes in the *A. thaliana* genome, the predicted *VQ* genes could number more than 100 in the *B. rapa* genome. However, in this study, we found that only 57 VQ genes were retained in the *B. rapa* genome, suggesting that there was extensive gene loss during genome duplication [32,33]. Similar cases were also reported in other *B. rapa* gene families, such as MADS-box [31], mitogen-activated protein kinase (MAPK) [34], WRKY [35], *etc*. Even so, the WGT event has indeed expanded the quantity of the *B. rapa* gene family members. We found 53 *BrVQ* genes showed a syntenic relationship with 28 *AtVQ* genes, implying that the numbers of duplicated *BrVQ* genes contain only approximately twice as many as that of the *AtVQ* genes. This result is consistent with a previous study which showed the triplicated *B. rapa* genome contains only approximately twice the numbers in that of *A. thaliana* because of genome shrinkage and differential loss of duplicated genes [36]. The expansion of the *BrVQ* gene family seems to mainly depend on the segmental duplication (41/57 genes; 71.9%), while one tandem duplicated pair (2/57 genes, 3.5%) may play a minor role. A similar phenomenon was reported in the *B. rapa* expansin superfamily which contains high segmental duplication (68%) and low tandem duplication (6.3%) [22]. Similarly, in the *A. thaliana* genome, a large proportion of gene families also fall into the low tandem and high segmental duplication class [37]. Besides these, gene duplication can generate gene functional redundancy, and these duplicate genes could develop divergent patterns of gene expression for stably maintaining through subfunctionalization [30,38]. For example, the two duplicated MADS-box genes, *BdMADS2* and *BdMADS4* from *Brachypodium distachyon*, displayed different expression patterns in all floral organs, and ectopic expression these two genes in Arabidopsis caused different phenotypic effects between them [39]. In our study, some paralogs showed different expression patterns, such as *BrVQ22-1*/*22-2*, *BrVQ24-1*/*24-2*, and *BrVQ30-1*/*30-2* in different tissues, and *BrVQ10-1*/*10-2*, *BrVQ11-1*/*11-3*, *BrVQ14-1*/*14-3*, and *BrVQ32-1*/*32-2* in various abiotic and hormone treatments, suggesting these *BrVQ* paralogs might be maintained through subfunctionalization. Additionally, a similar report was found in the duplicate NAC TF pairs from [40] and *AGAMOUS* (*AG*) genes from *Thalictrum thalictroides* [41].

### 3.2. Function of the VQ Proteins in Plant Growth and Development

Accumulating evidence has demonstrated that the transcription of the *VQ* genes is regulated by various endogenous and environmental signals, consistent with their diverse roles in plant growth and development [42]. The *IKU1*/*AtVQ14* gene is expressed preferentially in the early endosperm. *AtVQ14* mutation reduces endosperm growth and produces small seeds, suggesting the *AtVQ14* gene may be involved in seed development [7]. The loss-of-function mutation of *AtVQ14* results in a decrease of expression of *IKU2* (encoding a leucine-rich repeat kinase) and *MINI3* (encoding a WRKY family protein), both of which play an important role in seed development [43]. *AtVQ29* is expressed at a higher level in the stem than the root, rosette leaf, flower, and silique. Over-expression of *AtVQ29* causes hyposensitivity of hypocotyl growth to far-red and low-light conditions while its loss-of-function mutants display decreased hypocotyl elongation under the low intensity of far-red and white light, implying the VQ protein maybe involved in regulating plant seedling photomorphogenesis [8]. Moreover, over-expression of *AtVQ29* also substantially delays the blossom of the transgenic plant compared to the wild type [3]. *AtVQ8* was located in plastid. The recessive loss-of-function *AtVQ8* mutants exhibit pale-green and stunted-growth phenotypes throughout the entire life cycle, suggesting a predominant role in chloroplast development or photosystem assembly [3]. Besides these, over-expression of *AtVQ17*, *AtVQ18*, and *AtVQ22* also causes highly stunted growth [3]. In this study, we assessed the expression levels of the *BrVQ* genes in six Chinese cabbage tissues (Figure 7). The result showed that the majority of the genes were differentially expressed in the tissues that we analyzed. The *BrVQ* genes were expressed mainly in specific organs and tissues, suggesting that they may play important roles in the growth and development of these organs or tissues. Previous studies have shown that Gibberellin acid (GA_3_) has various regulation functions in high plants, such as simulating early seed development and organ growth and controlling fertilization time [44]. Thus, we also examined the expression profiles of the *BrVQ* genes in response to exogenous GA_3_ (Figure 9). The result showed expression of half the *BrVQ* genes was induced and some genes were up-regulated by more than two-fold. Besides these, similar expression patterns of *BrVQ* genes and their orthologs in *A. thaliana* were detected in different tissues. Taken together, these results indicate that the *VQ* gene family is extensively involved in plant growth and development.

### 3.3. Function of the VQ Proteins in Abiotic and Biotic Resistance

Drought, salt, heat, cold, and pathogen stresses are the main factors of reducing crop production. Many studies have been carried out to understand how tolerance to these stresses is regulated in plants. In the present study, we found that the majority of the *BrVQ* genes were induced by PEG_6000_ and NaCl treatments (Figure 8). Similar results were also found for the *VQ* genes in *Oryza sativa* and *Vitis* *vinifera* L. For instance, 22 *OsVQ* genes [4] and 18 *VvVQ* genes [6] were up-regulated by drought stress. However, studies also showed that the up-regulation of some *VQ* genes may have a negative effect on abiotic stress resistance. For example, *AtVQ9* expression is strongly induced by salt stress; however, the over-expression of *AtVQ9* rendered plants hypersensitive to salt stress [9]. *AtVQ15* was induced by dehydration and high salinity, whereas its over-expression lines exhibited an increased sensitivity to both salt and mannitol stresses during seed germination and seedling growth. On the contrary, the antisense lines were significantly more tolerant to these stresses [10]. We also found that the *BrVQ* genes were more responsive to heat and cold stresses, where the numbers of induced genes were more than those under PEG and NaCl stresses.

There are various stress perception and signaling pathways, some of which are special, but others may cross-talk [45]. Abscisic acid (ABA) is an important phytohormone and plays a critical role in the response to various abiotic stress signals [46]. Stress-responsive genes could be regulated by either the ABA-dependent or ABA-independent signaling pathway [45,47,48] and the ABA-dependent signaling pathway plays an important role in stress-responsive genes under osmotic stress [49]. The majority of the *BrVQ* genes either up- or down-regulated more than two-fold upon ABA stress compared to the untreated control (Figure 9). Similar results were also observed in the *OsVQ* genes upon ABA treatment [4]. In combined analysis of the expression patterns of *BrVQ* genes (Figure 8 and Figure 9), we found that some *BrVQ* genes displayed similar expression tendency under abiotic stress and ABA treatment. For example, *BrVQ1-2* was down-regulated at 3 h and followed by an increase at 24 h under PEG and ABA treatments. *BrVQ3-1* showed a down-regulation trend at 3 and 24 h under PEG, NaCl, and ABA treatments. The expression of *BrVQ22-2* exhibited similar accumulation trends during abiotic stresses and ABA treatment but *BrVQ10-2*/*10-3* showed the opposite tendency. These results suggested these *BrVQ* genes might be involved in stress response and ABA signaling. Salicylic acid (SA) is an important signal molecule that accumulates under abiotic and biotic stress [50]. We found that, after SA treatments (Figure 9), the expression of many *BrVQ* genes were up- or down-regulated compared to the untreated control. Similar results were observed in previous studies for other *VQ* genes. For example, 34 *AtVQ* genes are induced and differentially expressed in different tissues in response to SA treatment and pathogen infection [3]; 27 *OsVQ* genes are induced by at least one pathogen infection [4]. Sixteen *VvVQ* genes were induced by SA treatment [6]. *AtVQ21*/*MKS1*, as substrate of MPK4, is required for SA-dependent resistance in the *mpk4* mutants, and over-expression of *AtVQ21* in the wild-type plants is sufficient to activate SA-dependent resistance [11]. The *AtVQ23*/*SIB1* loss-of-function mutants compromised the induction of some defense-related genes by pathogen infection and SA treatments. However, over-expression lines increased the expression of defense-related genes upon pathogen infection and SA treatment [13]. Interestingly, *BrVQ21-1*/*21-2*/*21-3* and *BrVQ23-1*/*23-2*/*23-3* were up-regulated to a different extent after SA treatment, suggesting they might have similar regulation to that of *AtVQ21* and *AtVQ23*, respectively. Furthermore, we found that the responses of *VQ* genes in Chinese cabbage and Arabidopsis in the expression levels toward some abiotic stresses and hormone treatments were similar. Taken together, these results revealed that some VQ members could be actively involved in regulating plant responses to various abiotic and biotic stresses.

## 4. Experimental Section

### 4.1. Plant Materials, Growth Conditions, and Stress Treatments

Chinese cabbage cultivar “Guangdongzao” was used for all experiments. Plant seeds were sown in a glass Petri dish containing two wet filter papers. After germination, seedlings were transferred into pots (five seedlings in one pot) containing a growth medium with vermiculite and peat (3:1) and grown in a greenhouse at 20 ± 2 °C with a photoperiod of 16 h light and 8 h dark. Three-week-old seedlings were used for the abiotic and hormone treatments. For salinity and osmotic treatments, plant samples were irrigated with 200 mM NaCl and 20% (*w*/*v*) polyethylene glycol (PEG_6000_), respectively, until the solution flowed out from the bottom of the pot. For high and low temperature treatments, plant samples which were grown in the green house were transferred to an incubator at 35 °C or a refrigerating chamber at 4 °C, respectively. For planthormone treatments, we sprayed plant leaves with 200 μM Gibberellin A3 (GA_3_), 100 μM abscisic acid (ABA), and 200 μM salicylic acid (SA) solutions, respectively, until drops began to fall from the leaves. Then, the fully opened leaves of seedlings were harvested after 0, 3, and 24 h of the above abiotic and hormone treatments. For analysis of VQ gene expression in different tissues, plant organs were harvested after the plants bloomed; plant organs were harvested, including root (R), dwarf stem (DS), old leaf (OL), young leaf (YL), flower (FL), and flower bud (FLB), in three biological replicates for RNA preparation. All harvested samples were immediately frozen in liquid nitrogen and stored at −80 °C until use.

### 4.2. Sequence Retrieval

The VQ motif sequences are listed in the Pfam Database under the motif ID “PF05678” [51]. Chinese cabbage VQ motif–containing proteins were identified by using the local BLASTP in the *Brassica* database [17,32]. To confirm the presence of the VQ domain, the web tools from the Interpro program [52] and the SMART program [53] were used on the VQ proteins in *B. rapa*. The coding sequences (CDS) and amino acid sequences of the *B. rapa* VQ genes were downloaded from the *Brassica* database [17,32]. Thirty-four *AtVQ* gene and protein sequences from *A. thaliana* were retrieved from The *Arabidopsis* Information Resource (TAIR) [3,54]. Thirty-nine *OsVQ* gene and protein sequences from *O. sativa* were retrieved from the Rice Genome Annotation Project (RGAP) [4,55]. The homology searches between *A. thaliana* and *B. rapa* were performed by using the Gramene database [19] and the Basic Local Alignment Search Tool (BLAST) [56].

### 4.3. Identification and Analysis of the VQ Genes and Proteins in Chinese Cabbage

The physical locations of the *BrVQ* genes on the Chinese cabbage chromosomes were mapped by using Mapchart 2.2 (Plant Research International, Wageningen, The Netherlands). The BrVQ amino acid sequences were aligned by the software DNAMAN 6.0.40 (Lynnon Biosoft, Quebec, QC, Canada). Intron/exon structure analysis was performed by using the Gene Structure Display Server (GSDS) [57]. The protein size, molecular weight (*M*w), and theoretical isoelectric point (pI) were computed by using the ProtParam tool [58]. Phylogenetic tree was constructed with MEGA5 [59] on the basis of alignment with the full-length VQ protein sequences using the neighbor-joining method [60] with 1000 bootstrap replicates [61]. The distribution of the conserved motifs and domains were detected using the MEME suite [62] and the PlantsP database [20,63]. A MEME search was carried out with the following parameters: optimum motif width ≥6 and ≤300 and the maximum number of motifs set at 10.

### 4.4. RNA Isolation and qRT-PCR

Total RNA was isolated from each sample using a Trizol reagent (Invitrogen, Carlsbad, CA, USA) according to the manufacturer’s protocol. cDNA synthesis was carried out using a PrimeScript™ RT reagent kit with a gDNA Eraser (Takara, Dalian, China). Quantitative real-time PCR (qRT-PCR) was performed using a SYBR Green Master mix (Takara, Dalian, China) on an IQ5 Real-Time PCR Detection System (Bio-Rad, Hercules, CA, USA). The qRT-PCR primers for the *BrVQ* genes and *actin* gene are listed in Table S6. The *actin* gene was used as a constitutive expression control in the qRT-PCR experiments. Reactions were set up in a total volume of 20 μL containing 10μL of SYBR Green Master mix, 0.4 μL of each primer (10 µM), 7.2 μL of double-distilled water, and 2 μL of cDNA template. The PCR cycling conditions comprised an initial polymerase activation step of 95 °C for 1 min, followed by 45 cycles of 95 °C for 15 s, and 60 °C for 70 s. After each PCR run, a dissociation curve was plotted to confirm the specificity of the product and to avoid the production of primer dimers. Three replicates of each sample were conducted to calculate the average Ct values. The relative expression level was calculated by the comparative 2^−ΔΔ*C*t^ method [64]. Three biological replicates were carried out and the significance was determined with SPSS software (SPSS 17.0, IBM, Chicago, IL, USA) (*p* < 0.05).

## 5. Conclusions

We identified 57 BrVQ proteins by genome-wide identification, characterization, and expression analysis. Phylogenetic relationship analysis indicated that the VQ family in Chinese cabbage closely resembled that of *Arabidopsis*. Due to genome shrinkage and differential loss of duplicated genes during the WGT events, the number of the *BrVQ* genes is approximately twice of that of the *AtVQ* genes. The *BrVQ* genes were differentially expressed in six tissues as well as when the plants were exposed to various abiotic stresses and hormone stimulus. We found some similar expression patterns existing in *BrVQ* genes and their orthologs in *Arabidopsis*. Although some *AtVQ* genes have been functionally analyzed, other members of VQ remain to be further studied due to *VQ* gene structure diversification with the exception of the VQ motif. This information will provide a solid foundation for further functional studies of Chinese cabbage VQ proteins, and will be useful to better understanding the roles that *VQ* genes play in plant growth and development as well as mediating the cross-talk between abiotic stresses and hormone signaling.

## Supplementary Materials

Supplementary materials can be found at http://www.mdpi.com/1422-0067/16/12/26127/s1.
